# Dr. John Botsford Sabin

**Published:** 1911-06

**Authors:** 


Dr. John Botsford Sabin died at his home in Couer d'Alene, April 19, 1911, aged 65 years. He graduated from the University of Buffalo Medical Department in 1867.

